# Crosstalk between the circadian clock, intestinal stem cell niche, and epithelial cell fate decision

**DOI:** 10.1016/j.gendis.2025.101650

**Published:** 2025-04-18

**Authors:** Ji Liu, Zhihui Jiang, Juanmin Zha, Qiong Lin, Weiqi He

**Affiliations:** a Department of Gastroenterology, Children's Hospital of Soochow University, Jiangsu Key Laboratory of Neuropsychiatric Diseases and Cambridge-Suda (CAM-SU) Genomic Resource Center, Suzhou Medical College of Soochow University, Suzhou, Jiangsu 215123, China; b Department of Oncology, The First Affiliated Hospital of Soochow University, Suzhou, Jiangsu 215006, China; c Department of Gastroenterology, The Affiliated Wuxi Children's Hospital of Jiangnan University, Wuxi, Jiangsu 214023, China

**Keywords:** Cell cycle regulation, Circadian clock, Epithelial differentiation, Intestinal stem cell, Signaling pathways

## Abstract

The circadian rhythm, a 24-h cycle, plays a crucial role in regulating gut physiological processes, particularly the proliferation and differentiation of intestinal epithelial cells, which are essential for gut homeostasis and repair. This review discusses the complex interactions between circadian rhythms, cell cycle regulation, and key signaling pathways (Wnt, Notch, and Hippo) in the context of the intestinal stem cell niche and epithelial cell fate decisions. Key molecules such as brain and muscle ARNT-like 1 (BMAL1), circadian locomotor output cycles kaput (CLOCK), hairy and enhancer of split 1 (Hes1), and Yes-associated protein/transcriptional coactivator with PDZ-binding motif (YAP/TAZ) coordinate stem cell functions with circadian rhythms. We discuss how Notch signaling regulates the cell cycle and interacts with circadian rhythms. Additionally, we explore the role of Hippo-Wnt signaling in balancing cell proliferation and differentiation. Furthermore, we highlight the intricate relationships between circadian clock components and signaling pathways, emphasizing the importance of temporal coordination in determining epithelial cell fate. We also discuss shared enzymes, including casein kinase 1 delta (CK1δ), glycogen synthase kinase 3 (GSK3), and AMP-activated protein kinase (AMPK), which play a role in regulating the cell cycle, circadian rhythm, and signaling pathways. In summary, this review offers valuable insights into the regulatory mechanisms that control stem cell behavior and epithelial cell differentiation, suggesting promising directions for future research in intestinal biology and tissue homeostasis.

## Introduction

The circadian rhythm, a 24-h cycle, is controlled by key molecular players such as brain and muscle ARNT-like 1 (BMAL1) and circadian locomotor output cycles kaput (CLOCK). We explore the critical signaling pathways (Wnt, Notch, and Hippo) mediating the circadian clock's interaction and cellular processes. The Wnt pathway, known for its role in stem cell maintenance, is revealed as a central regulator that coordinates with cell cycle checkpoints, thus influencing epithelial cell fate decisions. Simultaneously, the Notch pathway acts as a temporal regulator, governing cell cycle events in sync with circadian rhythms. The Hippo pathway also plays a crucial role, balancing cell proliferation and differentiation in response to circadian oscillations. Furthermore, the relationship between Yes-associated protein/transcriptional coactivator with PDZ-binding motif (YAP/TAZ) and circadian regulators has been explored in detail, highlighting their role in directing epithelial cell fate within the circadian context. The coordination of these signaling pathways emphasizes the precise timing required for orchestrating cellular events within the intestinal microenvironment. In addition to these pathways, shared enzymes like casein kinase 1 delta (CK1δ), glycogen synthase kinase 3 (GSK3), and AMP-activated protein kinase (AMPK) are identified as key modulators of both the cell cycle and circadian rhythms, linking these processes with signaling pathways. This review aims to provide a comprehensive understanding of the dynamic crosstalk between the circadian clock and the cellular processes that regulate the intestinal stem cell niche and epithelial cell fate decisions.

## Intestinal epithelium structure and epithelial cell differentiation

**Figure 1:**
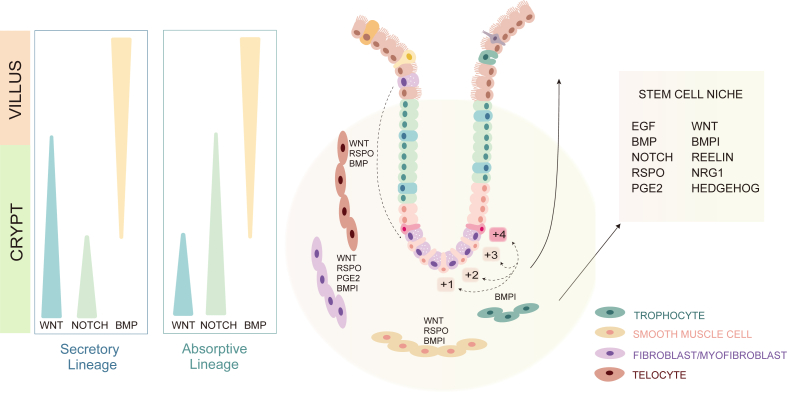
The signaling pathways involved in the intestinal stem cell niche and cell fate determination. Wnt, Notch, and BMP signals show spatial gradients along the crypt–villus axis. Lgr5^+^ crypt base columnar (CBC) cell and +4 stem cell pools located at the crypt base are surrounded by mesenchymal cells that produce and release niche-associated factors.

The intestinal epithelium is organized into crypts and villi. The villi, which decrease in length along the intestinal tract, project into the intestinal lumen and enhance absorption. Notably, the colon lacks villi, with the epithelial surface being relatively flat (Fig. 1). Intestinal stem cells reside at the base of the crypt, where they undergo rapid division and migrate upward toward the apex of the villi. The transit-amplifying cells, which have the progeny of stem cells, divide approximately 4–5 times in the crypt over 2 days before differentiating into mature epithelial cells. After maturation, these cells take around 3 days to reach the tip of the villi, where they undergo apoptosis and are shed into the intestinal lumen. The lifespan of a mature epithelial cell in the intestinal epithelium is approximately 5 days.^1^^,^^2^

## Intestinal stem cells

Intestinal stem cells can be divided into two populations: the Lgr5^+^ crypt base columnar (CBC) cells, located between Paneth cells, and the quiescent +4 stem cells. These stem cells play a dual role in self-renewal to maintain the stem cell pool or differentiation into progenitor cells, thereby contributing to lineage formation.^2^ CBC cells express markers such as leucine rich repeat containing G protein-coupled receptor 5 (Lgr5), olfactomedin 4 (Olfm4), tumor necrosis factor receptor superfamily, member 19 (Tnfrsf19, also known as Troy), achaete-scute family BHLH transcription factor 2 (Ascl2), SPARC related modular calcium binding 2 (Smoc2), ring finger protein 43 (Rnf43), and zinc and ring finger 3 (Znrf3).^3^ The +4 quiescent stem cells, also known as slowly cycling cells or DNA label retention cells, reside above Paneth cells and express markers like Bmi1, telomerase reverse transcriptase (Tert), HOP homeobox (Hopx), and leucine-rich repeats and immunoglobulin-like domains 1 (Lrig1) (Fig. 1).^3^ Under normal conditions, these +4 stem cells divide infrequently. However, during stress or injury, when Lgr5^+^ cells are depleted, the +4 stem cells play a critical role in the regenerative response.^3^ A newly identified subtype of quiescent cells, termed revival stem cells, has been found to express higher levels of clusterin, playing a pivotal role in injury-induced intestinal regeneration, a process regulated by Yes-associated protein 1 (YAP1).^4^

The differentiation of intestinal stem cells is regulated by several signaling pathways within the stem cell niche, including the epidermal growth factor (EGF), Wnt, bone morphogenetic protein (BMP), and Notch pathways.^5^ Paneth cells play a key role in the Wnt signaling pathway, as they secrete canonical Wnt ligands that interact with Frizzled receptors on neighboring stem cells. This interaction is crucial for maintaining the stem cell niche.^6^, ^7^, ^8^ Interestingly, senescent Paneth cells can regulate Wnt signaling by secreting the Wnt inhibitor Notum, which reduces Wnt activity.^9^ In contrast to mice, human Paneth cells lack expression of Wnt3, Wnt11, EGF, and R-spondin (Rspo), suggesting a more supportive role in the stem cell niche rather than being the primary drivers of Wnt signaling.^10^ In humans, Paneth cells also express markers such as Lgr5, SMOC2, ASCL2, and frizzled class receptor 9 (FZD9), indicating that they are more involved in receiving Wnt signaling rather than producing it.^10^

The intestinal stem cell niche is influenced by the surrounding mesenchymal cells, including fibroblasts/myofibroblasts, telocytes, trophocytes, and smooth muscle cells. These cells secrete various factors that shape the microenvironment of the stem cell niche.^11^ For instance, telocytes release WNT, RSPO, and BMP, while trophocytes secrete the BMP antagonist gremlin 1 (GREM1), which helps maintain Wnt signaling within the niche.^11^ Fibroblasts contribute WNT, RSPO, GREM1/2, and prostaglandin E2 (PGE2) to support stem cell function, while smooth muscle cells secrete WNT, RSPO, and GREM1/2. Additionally, neuregulin 1 (NRG1), which promotes the differentiation of secretory lineages in the human gut, is expressed by mesenchymal cells, macrophages, and Paneth cells. This protein plays a key role in epithelial development and repair.^11^, ^12^, ^13^ Recent studies have shown that the dynamics of Wnt signaling and extracellular matrix proteins, such as Reelin (RELN), are crucial for regulating the regenerative potential of intestinal stem cells.^14^

In some cases, there is a reversible transformation between Lgr5^+^ CBC cells and +4 stem cells.^5^ Remarkably, progenitor cells from both secretory and absorptive lineages can revert to a stem cell-like state.^15^ This plasticity in stem cell behavior suggests that stemness can be induced under certain conditions rather than being strictly predetermined.^16^ Research is ongoing to uncover the specific cell types and circumstances that trigger this reversion. It has been shown that most Lgr5^+^ intestinal stem cells express the p27^+^ marker, a sign of undifferentiated cells. Following injury, Lgr5^+^p27^+^ cells enter a G0 quiescent phase and inhibit the dedifferentiation of surrounding mature cells. This process is linked to transforming growth factor beta (TGFβ) signaling.^17^ Additionally, a unique dynamic stem cell movement has been observed in the small intestine, where cells migrate from the periphery of the crypt back toward the crypt base, facilitating faster regeneration compared with the colon.^18^

## Differentiated intestinal epithelial cells

**Figure 2:**
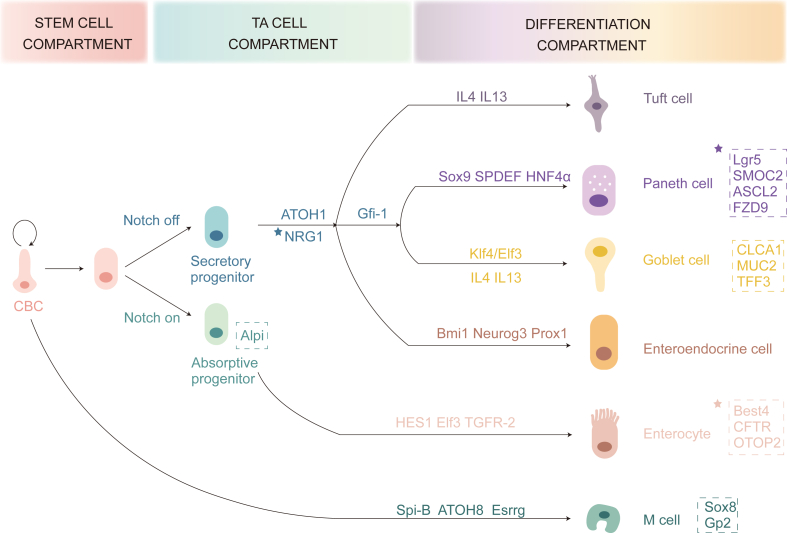
Types of intestinal epithelial cells with their specific expression (indicated by dashed boxes), along with the factors that initiate differentiation. The crypt–villus axis includes the stem cell compartment, transit-amplifying (TA) cell compartment, and differentiation compartment. Differentiated cell types comprise M cells, absorptive cells (enterocytes), and secretory cells (tuft cells, Paneth cells, goblet cells, and enteroendocrine cells). The “∗” symbol denotes specific expression to the human intestine.

After progenitor cells migrate along the crypt–villus axis, they differentiate into various cell types in the villi, including absorptive cells (enterocytes), secretory cells (goblet cells, enteroendocrine cells, Paneth cells, and tuft cells), and microfold cells (M cells). Absorptive cells, tuft cells, goblet cells, enteroendocrine cells, and M cells move toward the tip of the villi, where they are eventually shed into the intestinal lumen. In contrast, Paneth cells migrate toward the base of the crypt.^3^^,^^5^ The differentiation of cells into either absorptive or secretory lineages is primarily regulated by Notch signaling, which suppresses secretory cell differentiation while promoting the development of absorptive cells. This regulation is mediated by the induction of Hes1 expression, which represses atonal BHLH transcription factor 1 (Atoh1), a key factor in secretory cell commitment (Fig. 2).

### Absorptive cells

Absorptive cells, also known as enterocytes, are the most abundant cell type in the intestinal epithelium. They are tall and columnar and play a crucial role in nutrient absorption, including ions, water, glucose, peptides, and lipids. Enterocytes also contribute to the synthesis of secretory IgA, which targets antigens to defend against bacteria and viruses. The expansion of mucin production by goblet cells in the crypt region depends on calcium (Ca^2+^) transport facilitated by enterocytes.^19^ Notably, the activation of Notch signaling is essential for the development of absorptive cell precursors, as demonstrated by their expression of the *Alpi* gene. In mice, E74-like ETS transcription factor 3 (ELF3) is an upstream regulator of transforming growth factor beta receptor II (TGFBR2), playing a central role in the maturation of absorptive cells.^15^^,^^20^ Recently, a distinct subset of absorptive cells, marked by bestrophin 4 (BEST4^+^) expression, was identified at the apex of the crypts in the human gut.^21^ This population, which is absent in mice, works alongside goblet cells to enhance mucus production. BEST4^+^ cells in the small intestine express cystic fibrosis transmembrane conductance regulator (*CFTR*), while in the colon, they express otopetrin 2 (*OTOP2*), suggesting specialized roles in different parts of the intestine.^21^

### Secretory cells

Goblet cells, interspersed among absorptive cells, secrete mucus and are critical for protecting and lubricating the intestinal surface. These cells express markers like chloride channel accessory 1 (Clca1), mucin 2 (Muc2), and trefoil factor 3 (Tff3). Goblet cells also produce WAP four-disulfide core domain 2 (WFDC2), an antiprotease that inhibits bacterial growth, protects tight junctions, counters the commensal microbiota, and reduces mucosal inflammation. This secretion helps maintain the barrier homeostasis of the intestinal epithelium.^21^ Positioned near the exit of crypts, goblet cells with heightened quiescin sulfhydryl oxidase 1 (QSOX1) expression effectively secrete a mucus barrier.^22^

Paneth cells, located at the base of the crypts, secrete Wnt3a, EGF, and Notch ligands.^8^^,^^23^ Lactate, produced by the microbiota, stimulates the proliferation of intestinal stem cells, which leads to the expansion of Paneth and goblet cells.^24^^,^^25^ Interestingly, the absence of Paneth cells does not disrupt intestinal homeostasis, as other cells such as tuft cells, enteroendocrine cells, and mesenchymal cells can compensate for the loss of Paneth cells by providing alternative sources of Notch signaling.^11^^,^^23^ Paneth cells also contribute to the immune defense by secreting defensins and lysozyme, which help protect stem cells and strengthen the chemical barrier of the intestinal epithelium. The development and immune function of Paneth cells are influenced by interleukin (IL)22 and IL17a, which increase Paneth cell numbers and regulate the expression of antimicrobial peptides.^26^^,^^27^ Interestingly, the deletion of cyclin dependent kinase 5 (CDK5) regulatory subunit associated protein 3 (CDK5RAP3), a binding protein for CDK5 activator, results in the down-regulation of critical transcription factors like growth factor independent 1 (GFI1) and SRY-box transcription factor 9 (SOX9), which are essential for Paneth cell differentiation.^28^ Additionally, mature Paneth cells can undergo dedifferentiation and revert to a stem-like state following radiation exposure, a process regulated by Notch, phosphoinositide 3-kinase (PI3K)/protein kinase B (Akt), and Wnt signaling pathways.^29^, ^30^, ^31^, ^32^

Enteroendocrine cells play a vital role in the release of hormones such as glucose-dependent insulinotropic peptide, cholecystokinin, serotonin, somatostatin, peptide YY, and glucagon-like peptide-1 (GLP-1).^33^ These cells detect microbial metabolites and pathogen-associated molecular patterns (PAMPs) and secrete cytokines, contributing to the intestinal immune response. Key genes expressed in enteroendocrine cell precursors include *Bmi1* and prospero homeobox 1 (*Prox1*). Prox1^+^ cells are crucial for maintaining intestinal epithelial homeostasis and facilitating regeneration.^11^ Interestingly, the differentiation of human enteroendocrine cells requires the suppression of both Wnt and Notch signaling, while P38 mitogen-activated protein kinase (MAPK) inhibitors do not affect enteroendocrine differentiation.^33^

Tuft cells are a rare but distinct cell type found within the intestinal epithelium. They have a unique tubular-vesicular structure and a bundle of microfilaments, along with long microvilli extending into the lumen. These cells are usually sparse under normal conditions but proliferate during parasitic infections. Tuft cells produce antimicrobial peptides and express receptors such as toll-like receptor 4/5/9 (TLR4/5/9), which are involved in immune responses against parasites and bacteria.^34^ The differentiation of tuft cells is driven by the up-regulation of POU class 2 homeobox 3 (*Pou2f3*) in secretory progenitor cells. Additionally, tuft cells express components of the IL25 receptor, which suggests a positive feedback loop that amplifies IL25 signaling, potentially enhancing the immune response.^10^

### M cells

M cells, characterized by their “microfold” architecture (absence of apical microvilli), are mainly located in gut-associated lymphoid tissues, such as Peyer's patches in the ileum. M cells play a key role in antigen uptake from the intestinal lumen, which is then transported to the subepithelial region. These cells interact with macrophages to present antigens to lymphocytes, thus stimulating immune responses. Furthermore, M cells interact with B cells to enhance their functional maturation via CD137 signaling.^35^ The differentiation of M cells is regulated by receptor activator of nuclear factor-κB (RANK) ligand (RANKL), which activates the RANK receptor and leads to the expression of the SPIB transcription factor.^36^ The epigenetic regulator polycomb repressive complex 2 (PRC2) controls M cell development by modulating the expression of atonal BHLH transcription factor 8 (*Atoh8*) and estrogen-related receptor gamma (*Esrrg*).^37^^,^^38^ In mice, M cells express SRY-box transcription factor 8 (SOX8), which regulates glycoprotein 2 (*Gp2*) expression, a marker of functional maturation.^39^

## Intestinal stem cell niche and epithelial fate signaling

Several signaling pathways are intricately involved in regulating the intestinal stem cell niche and epithelial differentiation. Among these, the Wnt signaling pathway plays a central role in maintaining stem cell populations and guiding progenitor cells toward various differentiation fates. The Notch signaling pathway, on the other hand, is critical in determining whether cells differentiate into absorptive or secretory lineages. Additionally, the BMP pathway is vital for the maturation of secretory lineages. The coordinated actions of the Hippo, Hedgehog (Hh), and MAPK pathways also significantly influence both the intestinal stem cell niche and epithelial differentiation. This complex network of pathways ensures tissue homeostasis and supports the regenerative capacity of the intestine.

## Wnt signaling pathway

The Wnt signaling pathway involves several components: extracellular Wnt ligands, membrane receptors (Frizzled and LDL receptor related protein 5/6 (LRP5/6)), cytoplasmic elements (β-catenin, Dishevelled (DVL), GSK3, AXIN, adenomatous polyposis coli (APC), CK1), and nuclear factors (β-catenin translocation, TCF/LEF transcription complexes).^40^ In the absence of Wnt signaling, a “destruction complex” consisting of APC, CK1, GSK3, DVL, and AXIN phosphorylates and degrades β-catenin.^1^ However, when Wnt ligands bind to Frizzled and LRP receptors, this degradation is inhibited, leading to an increase in cytoplasmic β-catenin, which then translocates to the nucleus. In the nucleus, β-catenin interacts with TCF/LEF transcription factors to activate downstream Wnt target genes such as *c-Myc*, *Cyclin D1*, *Survivin*, *Axin2*, and matrix metalloproteinase 7/9 (*Mmp7/9*).^1^ Wnt signaling operates through both canonical and noncanonical branches, which regulate distinct cellular processes. Canonical Wnt signaling regulates cell proliferation, while the noncanonical pathway governs cell polarity and migration. For example, in *Drosophila* intestinal epithelium, noncanonical Wnt signaling coordinates stem cell migration to injury sites.^41^ Noncanonical Wnt signaling, primarily active in the upper crypt, promotes differentiation rather than proliferation and directs stem cells toward secretory fates, including Paneth or enteroendocrine cells.^42^

Recent discoveries have identified new regulators in the Wnt pathway, such as the CREPT protein, which is linked to tumorigenesis and acts as an activator of Wnt signaling. This protein helps control the homeostasis of Lgr5^+^ CBC cells.^43^ Wnt signaling is activated in response to intestinal damage, promoting cell proliferation and epithelial repair. However, excessive accumulation of Paneth cells in the stem cell niche can create a favorable environment for adenoma cells, leading to dysregulated Wnt signaling and contributing to colon cancer.^40^ Additionally, Wnt signaling plays a pivotal role in the differentiation of secretory lineages, particularly in promoting Paneth cell differentiation.^44^ Elevated levels of Wnt antagonists like Dickkopf-1 (DKK1) can result in the loss of various secretory cell types. Intestinal macrophages within the CD206^+^ subset secrete Wnt ligands, which help maintain the mesenchymal niche necessary for Paneth cell differentiation.^25^

## Notch signaling pathway

Notch signaling plays a crucial role in determining the fate of progenitor cells within the intestinal crypt. It is activated in the upper crypt, where Notch ligands (Jagged1/3 and Delta-like 1/3/4 (Dll1/3/4)) produced by secretory progenitors bind to Notch receptors (Notch1/2/3/4) on adjacent cells. This interaction triggers the cleavage of the Notch intracellular domain (NICD) by a disintegrin and metalloproteinase domain 10 (ADAM10) and γ-secretase. NICD then translocates to the nucleus, where it interacts with recombination signal binding protein for immunoglobulin kappa J region (RBPJ) and coactivators like Mastermind to activate transcription of target genes, including *Hes*, *P21*, and *Olfm4*. Notch signaling is involved in a process called “lateral inhibition”, where it prevents adjacent progenitor cells from differentiating into secretory cells. Notch signaling inhibits the expression of *Atoh1*, a key gene in secretory cell differentiation, by promoting the expression of *Hes1*. Conversely, inhibition of Notch signaling increases *Atoh1* expression, fostering differentiation into secretory cells. Atoh1^+^ cells can regain stem-like properties, participating in epithelial repair after injury.^45^^,^^46^ In addition, IL-17RA signaling in Atoh1^+^ cells is critical for secretory cell regeneration following injury. The regulation of *Atoh1* expression is a key factor in controlling the balance between absorptive and secretory cell differentiation.^47^ Goblet cell differentiation is regulated by factors such as Gfi1, Krüppel-like factor 4 (Klf4), or Elf3, while Paneth cell differentiation is directed by Gfi1, SAM-pointed domain-containing ETS transcription factor (Spdef), and Sox9.^8^ Enteroendocrine cell differentiation is influenced by neurogenin 3, which biases stem cell differentiation toward the secretory lineage.^48^ The simultaneous inhibition of Wnt, Notch, and MAPK signaling in intestinal organoids has been shown to significantly enhance the differentiation of enteroendocrine cells.^49^

Interestingly, depletion of Golgi membrane protein 1 (GOLM1/GP73) in the intestinal epithelium has been shown to abnormally activate Notch signaling, leading to impaired differentiation and maturation of the intestinal epithelium in mice.^50^ Inflammatory cytokines also play a regulatory role in Notch signaling and affect stem cell differentiation. For example, IL-33 suppresses Notch signaling in epithelial precursor cells, promoting their differentiation into Paneth and goblet cells.^51^ Additionally, Paneth cells activate CBC cells via Dll1 and Dll4 ligands, driving Notch signaling and supporting CBC cell proliferation.^52^ Notch signaling is essential for intestinal self-renewal and proliferation, particularly during recovery from injury. For instance, ghrelin has been shown to activate Notch signaling, aiding epithelial repair.^53^ In contrast, the loss of Notch 1/2 reduces epithelial cell proliferation following radiation-induced damage.^11^

## TGFβ/BMP signaling pathway

Bone morphogenetic proteins (BMPs) are secreted by mesenchymal cells in the crypt and villus regions, where they promote epithelial cell maturation. Myofibroblasts and smooth muscle cells at the crypt base secrete BMP antagonists such as gremlin 1/2, chordin-like 1, and noggin.^11^ BMP ligands bind to type II BMP receptors (BMPRII), leading to the activation of type I BMP receptors (BMPRI), which then phosphorylate R-Smads (Smad1/5/8). These R-Smads form complexes with co-Smad (Smad4), which migrate to the nucleus and regulate gene transcription. Unlike Wnt signaling, which is concentrated in the crypt, BMP signaling gradually increases along the crypt–villus axis. BMP signaling acts as a negative regulator of intestinal stem cell self-renewal and proliferation, preventing intestinal hyperplasia. Mice lacking BMPR1a in the intestinal epithelium exhibit increased proliferation of stem and progenitor cells, leading to the formation of intestinal adenomas.^1^^,^^5^^,^^54^ BMP signaling thus plays an important role in regulating Wnt signaling and maintaining the balance of stem cell self-renewal.^2^

## Hippo signaling pathway

The Hippo signaling pathway is centered around mammalian Ste20-like kinase 1/2 (MST1/2) and large tumor suppressor 1/2 (LATS1/2), which are activated by upstream cues such as Ras association domain family member 1–6 (RASSF1–6). Phosphorylation of MST1/2 and Salvador homolog-1 (SAV1) leads to the activation of LATS1/2 and Mps one binder 1 (MOB1).^55^ Downstream effectors of this pathway include YAP and WW domain-containing transcription regulator 1 (WWTR1, also called TAZ). Phosphorylation by LATS1/2 traps YAP/TAZ in the cytoplasm, preventing their interaction with transcriptional enhanced associate domain (TEAD) transcription factors. This curtails the activation of target genes.^56^ YAP and TAZ play a dual role in intestinal epithelial renewal. In cooperation with TEAD, they stimulate stem cell proliferation, while in conjunction with KLF4, they promote goblet cell differentiation.^57^ MST1/2 and LATS1/2, by limiting YAP/TAZ activity, are essential for preserving Wnt signaling and stem cell function.^58^ YAP deficiency impairs villus regeneration, indicating its critical role in maintaining epithelial integrity.^59^ RASSF1A is a tumor suppressor that regulates early cell fate determination and promotes differentiation by fostering the YAP-p73 transcriptional program.^60^ Interestingly, PGE2, secreted by mesenchymal fibroblasts, activates YAP, thus promoting epithelial regeneration.^61^

## Hedgehog signaling pathway

The Hh signaling pathway plays a critical role in the stem cell niche. Intestinal epithelial cells express Hh ligands, including Indian Hedgehog (Ihh) and Sonic Hedgehog (Shh), which bind to the Patched1/2 (PTCH1/2) receptor on mesenchymal cells. This interaction activates the Smoothened (Smo) receptor, which in turn stabilizes glioma-associated oncogene (Gli) transcription factors.^62^ Hh ligands modulate mesenchymal behavior, promoting the secretion of factors like WNTs, BMPs, and inflammatory mediators that regulate epithelial homeostasis.^62^ Hh signaling is crucial for maintaining the stem cell niche and regulating epithelial cell proliferation. It supports the expansion of mesenchymal cells and helps maintain epithelial homeostasis, preserving the crypt–villus axis.^63^ Reduced Hh signaling culminates in enlarged stem cell populations and heightened epithelial cell proliferation.^62^ In a reciprocal interaction, Wnt signaling directly regulates Shh gene expression in epithelial cells, which, in turn, influences mesenchymal cells to support villus formation.

## MAPK signaling pathway

MAPK signaling pathway plays a key role in determining the fate of goblet and Paneth cells by modulating Wnt signaling. In particular, the Shh2-mediated MAPK pathway influences the differentiation of these secretory cells. Growth factors and cytokines activate MAPK signaling through Src homology 2 domain-containing phosphatase 2 (Shp2), which regulates Ras/mitogen-activated protein kinase kinase 1 (Mek1)/MAPK activity. Suppression of MAPK signaling increases Wnt activity, promoting the generation of Paneth cells and stem cells, while elevated MAPK signaling reduces Paneth cells and favors differentiation toward goblet cells.^64^ At the crypt base, the Wnt signaling pathway suppresses the MAPK signaling pathway to maintain the stem cell pool.^65^ Deletion of Erk1/2 in epithelial cells disrupts differentiation into secretory cell lineages and impairs the proliferation or maturation of mesenchymal cells. Additionally, the MAPK signaling pathway plays a critical role in coordinating interactions between epithelial and mesenchymal components.^66^

## Circadian clock regulation in the gut

### Circadian clock

The hypothalamic suprachiasmatic nucleus serves as the main pacemaker for the circadian rhythm system, synchronizing daily physiological processes across organisms.^67^ This 24-h cycle coordinates various biological activities, including sleep–wake cycles, body temperature, immune responses, and metabolism regulation.^68^, ^69^, ^70^ Disruptions in the circadian rhythm due to jet lag, shift work, sleep disorders, or nighttime light exposure have been linked to metabolic diseases (such as obesity, diabetes, and nonalcoholic fatty liver disease) and chronic inflammatory conditions (like rheumatoid arthritis and asthma).^68^, ^69^, ^70^, ^71^ Additionally, circadian disruptions can alter the tumor immune environment, increasing the risk of several cancers, including prostate, breast, colon, and lung cancer.^72^, ^73^, ^74^, ^75^, ^76^

### Molecular mechanisms of the circadian clock

The circadian clock functions through an intricate feedback mechanism known as the transcriptional translation feedback loop. This loop involves key proteins such as BMAL1, CLOCK, period circadian regulator 1/2/3 (PER1/2/3), cryptochrome circadian regulator 1/2 (CRY1/2), nuclear receptor subfamily 1 group D member 1/2 (NR1D1/2, also called REV-ERBα/β), RAR-related orphan receptor A/B/C (RORα/β/γ), albumin D-box binding protein (DBP), and nuclear factor interleukin-3-regulated (NFIL3, also called E4BP4).^77^^,^^78^ BMAL1 and CLOCK form a complex that moves from the cytoplasm to the nucleus, binding to specific DNA sequences (E-BOX) to activate genes including *Per*, *Cry*, *Ror*, *Rev-Erb*, and *Dbp*. Regulation of PER and CRY in the cytoplasm involves phosphatases, E3 ubiquitin ligases, and kinases such as CK1δ/ε and AMPK. When PER and CRY enter the nucleus, they inhibit the activity of BMAL1-CLOCK, forming a negative feedback loop.^74^^,^^78^, ^79^, ^80^ A secondary loop involves RORs, REV-ERBs, and NFIL3, which regulate *Bmal1* transcription and compete for binding to ROR/REV-ERB-response element (RORE), helping to maintain rhythm in gene expression.^68^^,^^80^, ^81^, ^82^ These coordinated feedback loops ensure the circadian regulation of various physiological processes.^83^

### The central and gut clocks

The central clock, located in the hypothalamic suprachiasmatic nucleus, is the primary regulator of circadian rhythms, adjusting bodily functions based on light exposure over a 24-h period. Other “peripheral clocks” exist in tissues such as the liver, kidney, skin, small intestine, lung, pancreas, ovary, heart, and adipose tissue.^83^^,^^84^ Signals such as light/dark cycles, physical activity, and food intake synchronize these peripheral clocks via the autonomic nervous system and neuroendocrine signals.^69^^,^^80^^,^^81^ The “gut clock” specifically refers to circadian rhythms in intestinal epithelial stem cells and their differentiation, highlighting the connection between circadian rhythms and intestinal epithelial regeneration.^86^ Intestinal stem cells, located within crypts, exhibit circadian activity patterns influenced by light cycles and eating habits. These rhythms are crucial for determining the timing of stem cell division and the replacement of senescent cells. Any disruption in these molecular clock genes, due to inconsistent sleep or eating schedules, can impair stem cell activity and reduce the regenerative capacity of the gut. Circadian rhythms also regulate epithelial cell differentiation, leading to daily variations in epithelial cell types.^87^ The intricate crosstalk between the gut clock, intestinal epithelial stem cells, and epithelial differentiation is crucial for preserving gut homeostasis and overall health. Disruptions in circadian rhythms can destabilize epithelial balance, adversely affecting nutrient absorption, pathogen defense, and barrier integrity. A deeper understanding of the function of the gut clock in regulating intestinal epithelial stem cells and differentiation may lead to the development of innovative strategies for improving gut health and managing related disorders.

## Circadian-IEC crosstalk in proliferation and differentiation

### Coupling between cell cycle and circadian clock

**Figure 3:**
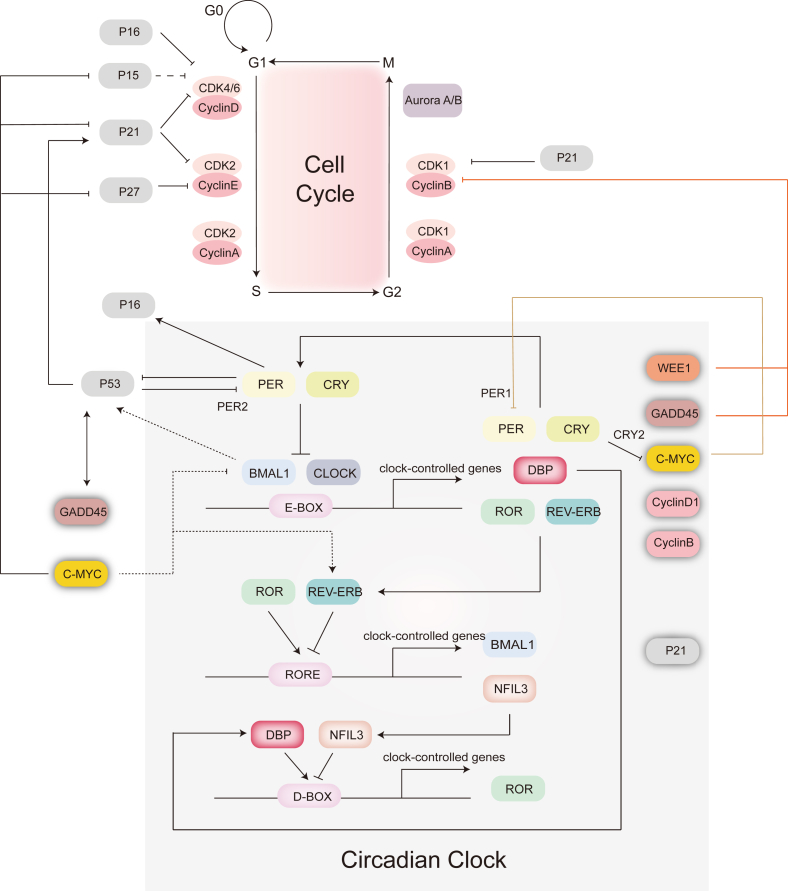
A schematic representation of the bidirectional relationship between the cell cycle and the circadian clock. Genes such as *Wee1*, *Gadd45*, *c-Myc*, *Cyclind1*, *Cyclinb*, *P21*, and *P16* are directly or indirectly related to the circadian clock and significantly impact the cell cycle. Wee1 and GADD45 inhibit cyclin B-CDK1, whereas c-Myc inhibits p15/p21/p27. P16 inhibits cyclin D–CDK4/6, and p21 inhibits cyclin B–CDK1, cyclin D–CDK4/6, and cyclin E–CDK2. Cyclin D1 and cyclin B are active during the G1/S and G2/M phases, respectively. BMAL1/CLOCK target gene *c-Myc* regulates key clock proteins, including PER1, BMAL1, and REV-ERB. The link between the circadian clock and the cell cycle involves p53, which activates GADD45A and itself. Activation, inhibition, and potential regulation are denoted by arrows, blunt ends, and dashed lines, respectively.

The cell cycle plays a vital role in mitosis and epithelial renewal, which is regulated through the interaction of cyclin–cyclin dependent kinase (CDK) complexes, including cyclin–CDK4/6, cyclin E–CDK2, cyclin A–CDK2 (G1/S), cyclin A–CDK1, cyclin Y–CDK14/16, and cyclin B–CDK1 (G2/M). During cellular stress, proteins like P16, P21, P27, P15, and WEE1 act as cell cycle inhibitors, whereas cell division cycle 25 (CDC25) activates cyclin B/CDK1, accelerating the cycle. The interaction between the circadian clock and cell cycle is evident, as the G2/M checkpoint kinase WEE1 exhibits rhythmic expression directly regulated by BMAL1/CLOCK (Fig. 3).^87^ ROR and REV-ERB have opposing effects on the expression of *P21*,^88^ and mutations in BMAL1 or CLOCK can lead to increased P21 levels and G2/M cell cycle arrest, which has implications for diseases like cancer.^89^ Similarly, activation of P16 by PER1/2 links the circadian clock with the regulation of G2/M and G1/S checkpoints.^90^ Disruption of the *CRY2* gene affects P53 expression, promoting tumor cell proliferation.^91^ BMAL1 binding to the P53 promoter initiates transcriptional activation of downstream tumor-suppressive pathways.^92^ Additionally, cell cycle regulators such as C-MYC, cyclin D1, and cyclin B are under the transcriptional control of BMAL1/CLOCK.^93^^,^^94^ C-MYC influences BMAL1 and CLOCK activity, inducing REV-ERB expression via direct binding, which can potentially alter circadian rhythm dynamics.^74^ Overexpression of *c-Myc* further inhibits BMAL1/CLOCK-induced activation of PER1, disrupting normal circadian regulation.^93^ Collaboratively, CRY2 and F-box and leucine-rich repeat protein 3 (FBXL3) target C-MYC for degradation, affecting its stability.^95^ The cell cycle also reciprocally impacts the circadian clock, as P53 interferes with BMAL1/CLOCK binding to the *Per2* promoter, thereby suppressing *Per2* expression.^96^

Rhythmic fluctuations in mitosis have been observed in epidermal cells,^2^ and circadian variations in the cell cycle have also been documented in the neurogenic niche of the adult zebrafish brain.^97^ Initially, intestinal epithelial proliferation was believed to lack rhythmicity. However, further studies have revealed a subtle proliferation rhythm in the intestinal epithelium under normal conditions.^98^ During inflammation-induced injury, BMAL1 plays a significant role in regulating intestinal epithelial cell proliferation, demonstrating a pronounced rhythmic pattern.^99^ Paneth cells contribute to this regulation by producing WNTs rhythmically, serving as pacemakers of the peripheral circadian clock in the intestinal epithelium. They regulate the timing of cell division for intestinal stem cells and progenitor cells across various lineages. Notably, the gene *cryptdin-1*, specific to Paneth cells, exhibits daily rhythmic expression.^6^ In the intestinal context, the Wnt signaling pathway acts as a critical mediator in the interaction between circadian rhythms and the cell cycle. Other pathways, such as Notch and Hippo, also participate in this intricate network, collectively influencing intestinal epithelial differentiation and emphasizing their essential role in maintaining tissue homeostasis.

## Coupling of Wnt signaling, cell cycle, and circadian clock

### Wnt signaling and cell cycle

**Figure 4:**
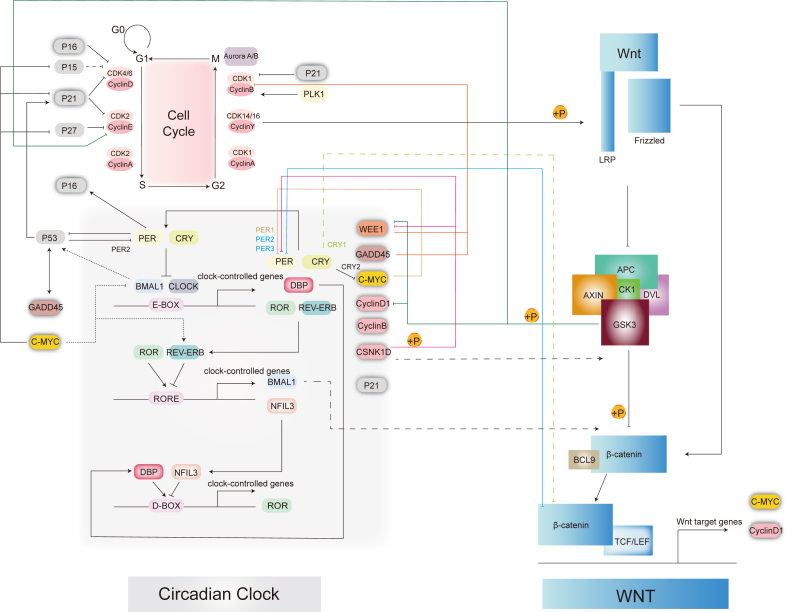
A schematic illustration of interactions among the Wnt signaling pathway, circadian clock, and cell cycle. Wnt target genes such as *c-Myc* and *CyclinD1* play essential roles in both the circadian clock and cell cycle. Key Wnt signaling components, including GSK3 and β-catenin, regulate clock proteins and cyclins. The circadian clock can influence Wnt signaling through CSNK1D, BMAL1, and CRY. Cyclin Y phosphorylates LRP6, linking cell cycle activation with Wnt signaling. Activation, inhibition, and potential regulatory relationships are represented by arrows, blunt ends, and dashed lines, respectively.

In the absence of Wnt signaling, GSK3 and CK1 phosphorylate β-catenin, marking it for ubiquitination and subsequent proteasomal degradation through the β-transducin repeats-containing protein (β-TrCP)/S-phase kinase-associated protein (Skp) pathway. When WNT ligands bind to FZD and LRP6 receptors, this activation blocks the destruction complex, allowing β-catenin to accumulate and translocate to the nucleus. In the nucleus, β-catenin forms a Wnt enhancer complex with B-cell lymphoma 9 (BCL9), driving the transcription of target genes such as *c-Myc* and *cyclin D1* (Fig. 4). Wnt signaling also significantly influences the cell cycle. For instance, GSK3 phosphorylates and inhibits Wee1, cyclin D1, and cyclin E, slowing the G1/S phase while accelerating the G2/M phase. CK1δ (CSNK1D) phosphorylates and degrades WEE1, further promoting progression through the G2/M phase. Moreover, the Wnt target gene *c-Myc* plays a key role in suppressing cell cycle inhibitors during the G1/S transition, leading to a shortened G1 phase and rapid cell division in stem cells. The cell cycle, in turn, impacts Wnt signaling. During mitosis, cyclin Y acts as a regulator by recruiting CDK14/16 to the plasma membrane, facilitating LRP6 phosphorylation, and activating Wnt signaling in the G2/M phase.^100^ Wnt signaling is also involved in critical processes such as chromosome arrangement, separation, and spindle localization, further highlighting its integration with cell cycle dynamics.^101^

### Wnt signaling and circadian clock

During stem cell division, BMAL1 and PER2 are significantly inhibited, allowing stem cells to bypass the cell cycle suppression typically imposed by the circadian clock.^102^ Crypt formation demonstrates rhythmicity, and the absence of Per1/2 disrupts this regulation, resulting in a reduction in small intestinal crypts.^102^ The circadian clock also modulates Wnt signaling, which is highly active in intestinal stem cells and progenitor cells at the crypt base, whereas circadian rhythm influence is weaker in this region.^6^ Interestingly, the circadian protein CK1δ (CSNK1D) activates Wnt signaling and has been implicated in promoting liver cancer progression.^103^ BMAL1 mutants exhibit reduced expression of *Lgr5* and *Hopx* in the colon epithelium, indicating impaired intestinal epithelial regeneration.^104^ BMAL1 and PER2 work together to suppress Wnt signaling by either down-regulating *RORα* or up-regulating *Rev-Erbα*.^105^ Enhanced expression of *Per2* or *Per3* inhibits tumor stem cell proliferation by suppressing Wnt signaling,^106^ while reduced PER3 levels stimulate Bmal1 expression and activate Wnt signaling.^107^^,^^108^ Furthermore, a decrease in endogenous Cry1 promotes β-catenin nuclear accumulation, enhancing Wnt activity.^109^ Disruptions in circadian rhythms can hyperactivate Wnt signaling, leading to increased c-Myc expression, which drives glycolytic metabolism and contributes to colorectal cancer development.^110^ Conversely, Wnt signaling can influence the circadian clock. For example, the Wnt target gene *c-Myc* suppresses *Bmal1* and *Per* expression while enhancing *Rev-Erbα* expression.^6^ Additionally, hepatocyte nuclear factor 4 alpha (HNF4A), an upstream regulator of Wnt3, inhibits BMAL1 and CLOCK transcriptional activity, helping to maintain tissue-specific circadian rhythms in colon cells.^111^ HNF4A thus serves as a crucial link between the circadian clock and the Wnt signaling pathway, which is closely tied to the intestinal peripheral circadian clock.

## Coupling of Notch signaling, cell cycle, and circadian clock

### Notch and Wnt signaling

The Wnt signaling pathway primarily suppresses the Notch signaling pathway through two key mechanisms. First, it involves DVL-mediated inhibition of Notch. Second, GSK3 phosphorylates the Notch intracellular domains, further suppressing its activity. However, under certain conditions, Notch signaling can reciprocally influence the Wnt pathway, either by inhibiting or activating it. For instance, during intestinal epithelial differentiation into absorptive lineages, activation of the Notch pathway suppresses Wnt signaling. Conversely, differentiation into secretory lineages activates the Wnt pathway, which, in turn, facilitates Notch activation through complex interactions.^112^

### Notch signaling and cell cycle

The Notch and Wnt signaling pathways share common target genes, such as *cyclin D* and *c-Myc*, both of which play crucial roles in accelerating the cell cycle.^113^ When Wnt and Notch pathways are activated simultaneously, they collaboratively drive rapid stem cell division. Conversely, suppression of the Notch pathway impairs cell proliferation; for example, inhibition using a γ-secretase inhibitor (DAPT) results in cell cycle arrest at the G0/G1 phase.^114^ Interestingly, Hes1-deficient pituitary cells show increased levels of *P27* and *P57*, suggesting that HES1 may contribute to the rapid proliferation of stem cells via the Notch pathway.^115^ Overexpression of NICD in chondrocytes similarly elevates P57 levels, indicating that P57 may provide negative feedback to regulate the cell cycle in cases of excessive Notch activity.^116^ Additionally, the Notch target gene *P21* functions as a negative regulator of cell proliferation.^117^ In mouse gastric chief cells, P57 is continuously expressed under steady-state conditions but decreases rapidly after injury, followed by overexpression during recovery. This fluctuation triggers dedifferentiation, transitioning chief cells into a stem cell state and establishing a reserve stem cell population. These observations underscore the potential role of P57 in dedifferentiation and the maintenance of stem cell plasticity.^118^

### Notch signaling and circadian clock

**Figure 5:**
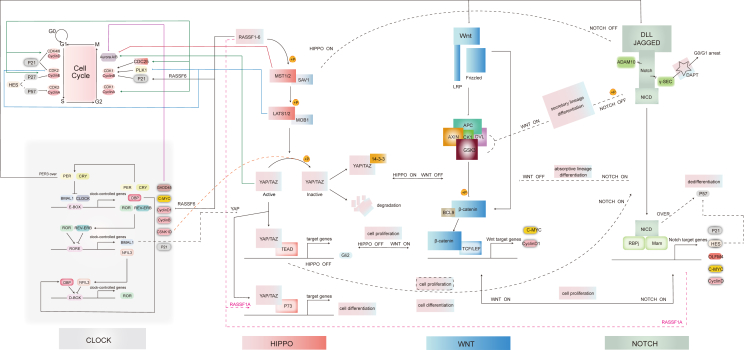
The interaction map of the Hippo, Wnt, and Notch signaling pathways and their crosstalk with the circadian clock and cell cycle. The schematic highlights how these signaling pathways cooperate or antagonize one other to regulate cell proliferation and differentiation. Activation, inhibition, and potential regulation are illustrated with arrows, blunt ends, and dashed lines, respectively.

The Notch downstream target gene *Hes1* exhibits rhythmic expression in various mouse cells and plays a key role in regulating stem cell proliferation and differentiation.^119^ HES1, similar to BMAL1/CLOCK, can bind to E-BOX sequences, enabling it to assist BMAL1/CLOCK in driving rhythmic regulation. This parallel may explain the observed reduction in Wnt signaling at the crypt apex, where Notch signaling operates in a circadian rhythm.^2^ Clock-associated genes, including *P21*, *Hes1*, *c-Myc*, and *cyclin D*, are downstream targets of Notch signaling, further emphasizing the impact of the circadian clock on epithelial differentiation (Fig. 5). In tumor stem cells, increased expression of *Per3* has been shown to inhibit both Notch and Wnt signaling, thereby reducing the self-renewal capacity of these cells. This highlights the significant interplay between the circadian clock and signaling pathways in regulating stem cell behavior and differentiation.^120^

## Coupling of Hippo signaling, cell cycle, and circadian clock

### Hippo and Wnt signaling

The activation of the Hippo signaling pathway results in the inactivation of YAP/TAZ, which suppresses Wnt target gene expression. In this way, Hippo signaling acts as a regulator that restrains Wnt activity, controlling stem cell proliferation and affecting Paneth cell migration. When Hippo activity is diminished, YAP/TAZ are activated, move to the nucleus, and interact with TEAD transcription factors to activate downstream genes. YAP/TAZ can also partner with β-catenin in the nucleus to stimulate Wnt target genes.^56^ Additionally, the upstream regulator RASSF induces Hippo activation through phosphorylation and promotes the binding of YAP/TAZ to P73, promoting goblet cell differentiation.^121^ This establishes an antagonistic relationship between the Hippo and Wnt pathways, particularly in regulating stem cell proliferation and regeneration. Active Hippo signaling restrains Wnt activity, dampening its effects, while reduced Hippo signaling enhances Wnt pathway activity, driving rapid stem cell proliferation. In this context, nuclear YAP/TAZ works with KLF4 and P73 to promote goblet cell differentiation. Simultaneously, enhanced Wnt signaling drives the differentiation of secretory cell lineages, highlighting the complementary roles of these pathways in maintaining intestinal epithelial balance.

### Hippo and Notch signaling

The interaction between the Hippo and Notch signaling pathways reveals a dynamic regulatory relationship. Activation of the Hippo pathway suppresses Notch signaling by phosphorylating and inhibiting YAP.^58^ Conversely, when Hippo signaling is attenuated, the subsequent activation of YAP/TAZ can enhance Notch signaling through transcriptional regulation.^57^ This interaction mirrors the antagonistic relationship observed between Hippo and Wnt signaling pathways. These interactions contribute to the synchronized regulation of rapid stem cell division, driven by the coordinated activity of Wnt and Notch pathways. Additionally, RASSF1A plays a dual role in this context. It promotes goblet cell differentiation while inhibiting Notch signaling by facilitating the ubiquitination of HES1.^122^ This dual function of RASSF1A reflects its critical role in directing stem cell differentiation away from absorptive cells and toward secretory lineages, further emphasizing its importance in maintaining epithelial cell diversity.

### Hippo signaling and cell cycle

The activation of the Hippo pathway results in the up-regulation of cell cycle inhibitors, leading to cell cycle arrest at the G1/S and G2/M phases. For example, RASSF6 inhibits *cyclin D1* and increases *P21* levels, which reduces Wnt signaling activity and thus stem cell proliferation.^123^ Conversely, when Hippo signaling is inhibited, YAP/TAZ become active, promoting cell cycle progression and stimulating Wnt-driven stem cell proliferation. Proteins like cellular communication network factor 1 (CCN1), produced by mesenchymal cells, enhance YAP activity and induce *Dkk1* expression, thereby supporting intestinal stem cell proliferation.^124^ The inhibition of YAP/TAZ by LATS proteins is controlled by the cell cycle, peaking during the G1 phase.^125^ In early embryos, stem cell differentiation begins with the activation of developmental genes during the G1 phase.^126^ Similarly, intestinal stem cell differentiation may also be decided during the G1 phase, with highly active YAP/TAZ promoting differentiation into goblet cells. The Hippo pathway also interacts with cell cycle checkpoints, such as the G1 tetraploidy checkpoint, mitotic spindle assembly checkpoint, and DNA damage checkpoint, to regulate cell division.^127^

### Hippo signaling and circadian clock

In mice lacking BMAL1 or with disrupted circadian rhythms, there is increased YAP/TEAD activity alongside weakened Wnt signaling, which makes intestinal tumor stem cells more prone to rapid proliferation.^85^ Eliminating BMAL1 has shown potential in preventing renal fibrosis by inhibiting *Gli2*, even in the context of reduced Wnt signaling.^128^ The circadian clock protein CK1δ (CSNK1D) is believed to enhance Hippo pathway activity while deactivating YAP/TAZ. Both the Hippo and Wnt pathways positively influence the circadian clock in *Drosophila* stem cells, affecting their differentiation into enteroendocrine cells.^87^ This is consistent with findings in mice, where *Bmal1* knockout reduces the number of enteroendocrine cells in the colon.^104^

### Coupling of cell cycle, circadian clock, and other signaling pathways

When the RAS pathway is abnormally activated, it suppresses BMAL1/CLOCK, leading to changes in circadian rhythm. The MAPK pathway is crucial for maintaining circadian rhythm and, when overactive, can contribute to tumor formation.^129^ Deletion of *Rev-erbα* up-regulates the MAPK pathway in chondrocytes.^130^ MAPK also regulates gene expression during the circadian cycle, particularly affecting genes activated by light at night.^131^ There is also bidirectional regulation between the Hh signaling pathway and the circadian clock in liver cells. Hh signaling genes show circadian changes, and the Ihh ligand in serum oscillates on a 24-h cycle. Deletion of *Bmal1* leads to changes in Hh expression, suggesting mutual regulation between these pathways.^132^

The BMP pathway also interacts with circadian mechanisms, as it inhibits *c-Myc* and promotes *P15* expression, mirroring its antagonistic effect on the Wnt pathway. Hh pathway targets include cyclin D/E and c-Myc, while the MAPK pathway influences CDK4/6, cyclin D1, P21, and P27, all of which are involved in the cell cycle and circadian regulation.^133^

### Shared enzymes connecting the circadian clock, cell cycle, and pathways regulating intestinal epithelial niche and differentiation

The interaction between the cell cycle and the circadian clock is influenced by key enzymes such as F-box/WD repeat-containing protein 7 (FBXW7), β-TrCP, CK1δ, GSK3, and AMPK.^134^ These enzymes, primarily through ubiquitination or phosphorylation, target multiple proteins across these processes, highlighting their multifaceted regulatory functions (Table 1).

**Table 1:** Shared enzymes connecting cell cycle, circadian clock, and signaling pathways in intestinal stem cell niche and epithelial cell differentiation. Shared enzymes include β-TrCP, CK1δ, GSK3, and AMPK, with roles in governing essential constituents of the cell cycle, circadian clock, and signaling pathways via ubiquitination or phosphorylation mechanisms. Upward arrows denote augmentation, while downward arrows indicate degradation. Additional outcomes, including stabilization or activation, are also indicated.

Enzyme	Cell cycle	Circadian clock	Signaling pathway
FBXW7 (ubiquitination)	CyclinE↓(G1/S transition)^135^ c-Myc↓^136^ CyclinL1↓(G2/M transition)^137^	REV-ERBΑ↓^138^ CRY2↓^139^	NOTCH2↓^140^ WNT/β-catenin↓^141^ NICD↓^52^
β-TrCP (ubiquitination)	WEE1↓^142^ CDC25A↓(accelerator)^143^ CEP68↓(centriole separation)^144^ PLK1 ↓(G2/M transition)^145^	PER1↓^146^ PER2↓^147^	β-catenin↓^148^ SMAD4↓^149^ YAP↓^150^ GLI↓^151^ NICD↓^52^
CK1δ (gene-CSNK1D) (phosphorylation)	WEE1↓^152^	PER↓^78^, ^79^, ^80^	YAP stabilization^150^ DVL3 stabilization/Wnt↑^103^ GLI activation^153^
GSK3 (phosphorylation)	WEE1↓^154^ CyclinD1↓^155^ CyclinE↓^156^	BMAL1 stabilization^157^	WNT↓^2^ NOTCH2 ICD activity↓^52^
AMPK (phosphorylation)	P27 stabilization^158^ CyclinY/CDK16 activation^159^	CRY↓^78^, ^79^, ^80^	YAP ↑^160^

FBXW7 mediates ubiquitination to regulate cell cycle transitions by degrading cyclin E, c-Myc, and cyclin L1.^135^, ^136^, ^137^ Concurrently, FBXW7 modulates circadian rhythms through the degradation of REV-ERBα and CRY2.^138^, ^139^ Additionally, FBXW7 modulates key signaling pathways, such as the WNT/β-catenin and NOTCH pathways, by degrading NOTCH2 and NICD, thus affecting intestinal epithelial differentiation.^52^^,^^140^^,^^141^ Similarly, β-TrCP facilitates ubiquitination to regulate the cell cycle by targeting WEE1, CDC25A, centrosomal protein 68 (CEP68), and polo-like kinase 1 (PLK1), proteins critical for cell cycle checkpoints and centriole separation.^142^, ^143^, ^144^, ^145^ It also plays a role in circadian rhythm by degrading PER1 and PER2, core clock proteins, thereby impacting the molecular clock.^146^, ^147^ Furthermore, β-TrCP is involved in signaling pathways by degrading β-catenin, SMAD4, YAP, GLI, and NICD, influencing WNT, TGFβ, Hippo, and Hh signaling networks, all of which are essential for maintaining epithelial cell homeostasis.^52^^,^^148^, ^149^, ^150^, ^151^ CK1δ, through phosphorylation, regulates both cell cycle and circadian rhythms by targeting WEE1 and PER proteins, respectively.^78^, ^79^, ^80^^,^^152^ This enzyme also stabilizes YAP and DVL3, thereby enhancing WNT signaling, and activates GLI, a key mediator of Hh signaling, further reinforcing its role in epithelial differentiation.^103^^,^^150^^,^^153^ GSK3 regulates the cell cycle by targeting WEE1, cyclin D1, and cyclin E for degradation.^154^, ^155^, ^156^ Its role in circadian regulation is evident through the stabilization of BMAL1.^157^ Additionally, GSK3 inhibits WNT signaling and reduces the activity of the intracellular domain of NOTCH2, linking its function to epithelial differentiation.^2^^,^^52^ AMPK, through phosphorylation, stabilizes P27 and activates the cyclin Y/CDK16 complex.^158^^,^^159^ It also modulates circadian rhythms by regulating CRY protein stability and enhances YAP activity, thereby contributing to the regulation of intestinal epithelial homeostasis.^78^, ^79^, ^80^, ^160^

## Conclusion and future direction

This review summarizes the complex connections between cell cycle regulation, signaling pathways, and circadian rhythms within the intestinal stem cell niche. The roles of Hippo, Wnt, and Notch pathways in controlling stem cell behavior and fate decisions are highlighted. Their interactions, as well as the effects of their activation or inhibition, provide insights into the regulation of intestinal epithelial cell differentiation and tissue regeneration. The link between the cell cycle and circadian clock is also explored, with a focus on enzymes like CK1δ, GSK3, and AMPK, which coordinate these processes. Future studies should investigate how the Hippo, Wnt, and Notch pathways converge to regulate stem cell fate. Understanding these mechanisms may lead to therapies for diseases caused by abnormal stem cell behavior. The timing and synchronization of the cell cycle and circadian clock require further exploration. Understanding the cross-talk between signaling pathways and the circadian clock may reveal new therapeutic targets and provide opportunities for precision medicine strategies. Additionally, advanced technologies, such as single-cell analysis and imaging, will help uncover the diversity and organization of cells in the intestinal niche, supporting the development of targeted therapies.

In summary, the Hippo, Wnt, and Notch pathways, together with the crosstalk of the cell cycle and circadian clock, are key regulators of intestinal stem cell behavior. Continued research will deepen our understanding of these mechanisms and enable new treatments for intestinal epithelial dysfunction.

## CRediT authorship contribution statement

**Ji Liu:** Conceptualization, Investigation, Methodology, Writing – original draft, Writing – review & editing. **Zhihui Jiang:** Investigation, Writing – original draft. **Juanmin Zha:** Investigation, Writing – original draft, Writing – review & editing, Funding acquisition. **Qiong Lin:** Conceptualization, Investigation, Writing – original draft, Writing – review & editing. **Weiqi He:** Conceptualization, Funding acquisition, Investigation, Methodology, Supervision, Validation, Writing – original draft, Writing – review & editing.

## Funding

This work is supported by the 10.13039/501100004608Natural Science Foundation of Jiangsu Province, China (No. BK20241919 to W.H.), the 10.13039/501100001809National Natural Science Foundation of China (No. 31971062 to W.H.), and the Health Promotion Project (China) (No. BJHA-CRP-084 to J.Z.). This work is also supported by the Senior Talent Research Program of “Western Medicine learning Traditional Chinese Medicine” in Jiangsu Province, China (to J.Z.), and the International Joint Research Center for Genomic Resources (China) (No. 2017B01012).

## Conflict of interests

All authors reported no conflict of interests in this work.
